# Impaired Hemorheology in Exacerbations of COPD

**DOI:** 10.1155/2017/1286263

**Published:** 2017-09-27

**Authors:** Erhan Ugurlu, Emine Kilic-Toprak, Ilknur Can, Ozgen Kilic-Erkek, Goksel Altinisik, Melek Bor-Kucukatay

**Affiliations:** ^1^Faculty of Medicine, Department of Pulmonology, Pamukkale University, Denizli, Turkey; ^2^Faculty of Medicine, Department of Physiology, Pamukkale University, Denizli, Turkey

## Abstract

**Background:**

Chronic obstructive pulmonary disease (COPD) is characterized by progressive airflow limitation. Cardiovascular-related comorbidities are established to contribute to morbidity and mortality especially during exacerbations. The aim of the current study was to determine alterations in hemorheology (erythrocyte aggregation, deformability) in newly diagnosed COPD patients and their response to medical treatment and to compare with values of COPD patients with exacerbations.

**Materials and Methods:**

The study comprised 13 COPD patients, 12 controls, and 16 COPD patients with exacerbations. The severity of COPD was determined according to* Global Initiative for Chronic Obstructive Lung Disease* guidelines. Red blood cell (RBC) deformability and aggregation were measured by an ektacytometer.

**Results:**

RBC deformability of COPD patients with exacerbations was decreased compared to the other groups. Erythrocyte aggregation and plasma fibrinogen of COPD patients determined during exacerbations were higher than control.

**Conclusion:**

Decreased RBC deformability and increased aggregation associated with exacerbations of COPD may serve as unfavorable mechanisms to worsen oxygenation and thus clinical symptoms of the patient. Treatment modalities that modify rheological parameters might be beneficial.

## 1. Introduction

Chronic obstructive pulmonary disease (COPD) is a common, preventable, and manageable disease process that is characterized by progressive persistent airflow restrictions and related to increased chronic inflammatory reaction of the airways and lungs against harmful gases and particles [1]. Exacerbations of COPD are defined as the worsening of symptoms such as increment of dyspnea, phlegm volume, and phlegm purulence, usually accompanied by hypoxia and hypercapnia [2, 3]. The most common causes of COPD exacerbations are infections. Inflammation is an important component of the disease [4]. Macrophages and neutrophils play an important role in the inflammatory process [5]. On the other hand, RBC were proposed as biosensors for the stadium of COPD [6]. Nowadays, COPD treatment options include quitting smoking, pharmacological treatment (short-acting anticholinergic, short-acting beta_2_-agonist, long-acting anticholinergic, long-acting beta_2_-agonist, and inhaled corticosteroids), and nonpharmacological treatment (pulmonary rehabilitation) [7–9]. Aim of pharmacological treatment is to improve the quality of life and exercise tolerance beside achieving the reduction of symptoms and attacks [7, 8]. Inhaled corticosteroids specifically reduce the number of COPD exacerbations [10]. In COPD exacerbation first of all, it is important to identify the etiology. Viral and bacterial infections, air pollution, and environmental factors can initiate COPD exacerbation. Thus, antibiotics and oral steroids are choices of treatment [11].

Hemorheology is a branch of biorheology that focuses on blood and its interactions in both macro- and microcirculation under the influence of the applied constraints [12]. Erythrocyte deformability, red blood cell (RBC) aggregation, hematocrit (Hct), and blood viscosity are well-known components of hemorheology [12–14]. The flow properties of blood play significant roles in tissue perfusion. These properties are influenced by pathophysiological processes, including a variety of pulmonary diseases, thereby increasing the clinical relevance of blood rheology information [13, 15–17]. In COPD patients, RBC were shown to alter morphologically, like cytoskeleton changes, ultrastructural modifications, and reductions of glycophorin A, band 3, and RBC thiols [7, 18, 19]. Rheological blood properties were demonstrated to progressively deteriorate as pulmonary insufficiency increases in patients with chronic bronchitis [18]. There are limited number of studies exploring hemorheological alterations in COPD [16, 20–22]. The results of these studies were obtained either from COPD patients at different stages of the illness and age [18, 20, 22] or from patients receiving different types of therapy (medication, exercise) [16, 20, 23]. Additionally, different types of measurement techniques, some being rather old, were used in a variety of the studies mentioned above, which makes it difficult to compare the results [20, 21, 24]. Thus, we aimed to determine the possible alterations in RBC deformability and aggregation in newly diagnosed COPD patients and their response to medical treatment and to compare these parameters with values of COPD patients with exacerbations for the first time in the literature. The results of the current study are expected to contribute to the formation of alternative treatment options for COPD.

## 2. Materials and Methods

### 2.1. Subjects

Newly diagnosed patients with COPD as well as COPD patients with exacerbations according to the Global Initiative for Chronic Obstructive Lung Disease (GOLD) criteria at the Pulmonology Department of Pamukkale University were enrolled in the study. The severity of COPD was evaluated by the GOLD, the American Thoracic Society, and the European Respiratory Society. All subjects underwent respiratory function testing performed with the patient seated. Forced vital capacity (FVC), forced expiratory volume in the first second (FEV1), forced expiratory flow 25% to 75% (FEF 25/75), and peak expiratory flow (PEF) were measured with a clinical spirometer. We considered the predicted percentage value of air exhaled during the first second of forced expiration (pred FEV1%) and the Tiffeneau index (FEV1/FVC). In particular, pulmonary function test was evaluated in 13 newly diagnosed COPD patients before and after 1 month of medical treatment and in 16 COPD patients with exacerbations. The control group consisted of 12 subjects with comparable age and gender distribution. Among these subjects who had hypoxia (oxygen saturation ≤ 97%) on a pulse oximeter reading applied via the finger, smokers and patients with other systemic diseases were excluded from the study. Patients with no obvious medical problem were included in newly diagnosed group. COPD exacerbation group consisted of severe and hospitalized patients. FEV1/FVC < 70% with an expected FEV1 > 80% indicates stage 1 (mild) of the disease process; 50% < FEV1 < 80% indicates stage 2 disease (moderate); 30% < FEV1 < 50% indicates stage 3 disease (severe); and FEV1 < 30% indicates stage 4 disease (most severe) [1]. After obtaining pretreatment blood samples, patients were treated according to the stage of the illness. Posttreatment blood samples were taken after 1 month of treatment. Although 40 newly diagnosed patients were included in the beginning, 13 of them completed the study. Ethical approval was obtained from Pamukkale University Ethical Board on Human Experiments, with the approval number 60116787-020/35881. All participants gave their written informed consent to the study.

Treatment was in accordance with recently published guidelines. Combination of short-acting beta_2_ agonists and/or short-acting anticholinergics was applied to stage I COPD patients, if necessary. Stage II patients were treated with long-acting anticholinergics for 1 month. COPD exacerbation group received antibiotics and oral or IV steroids.

### 2.2. Samples and Measurements

Venous blood samples of approximately 10 mL were drawn by venipuncture after 8 hours of fasting. Blood samples containing EDTA (1000/cs, 100/cp) were appropriately transported to the physiology laboratory and hemorheological tests were performed within 3 hours in accordance with “new guidelines for hemorheological laboratory techniques” [25]. Hematological parameters as complete blood count were determined by an electronic hematology analyzer (Siemens ADVIA® 2120i System, Siemens Healthcare Diagnostics, Japan). Sodium citrated blood samples were analyzed for plasma fibrinogen concentration using a fully automated coagulometer [Beckman COULTER Acl top 700, instrumentation laboratory (USA)].

### 2.3. Erythrocyte Deformability Measurements

RBC deformability was determined at various fluid shear stresses by laser diffraction analysis using an ektacytometer (laser assisted optical rotational cell analyzer (LORCA), RR Mechatronics, Hoorn, Netherlands). The system has been described elsewhere in detail [26]. Briefly, a low Hct suspension of RBC in an isotonic viscous medium (4% polyvinylpyrrolidone 360 solution; MW 360 kD; Sigma P 5288; St. Louis, MI) was sheared in a Couette system composed of a glass cup and a precisely fitting bob, with a gap of 0.3 mm between the cylinders. A laser beam was directed through the sheared sample, and the diffraction pattern produced by the deformed cells was analyzed by a microcomputer. On the basis of the geometry of the elliptical diffraction pattern, an elongation index (EI) was calculated as EI = (*L* − *W*)*/*(*L* + *W*), where *L* and *W* are the length and width of the diffraction pattern, respectively. EI values were determined for 9 shear stresses between 0.3 and 30 Pa and similar patterns of RBC deformability alterations were obtained between groups at all stress levels. All measurements were carried out at 37°C. Subsequently, the shear stress for half-maximal deformation (SS1/2) was calculated using this nine-point data set for each measurement by employing a Lineweaver–Burk analysis as described elsewhere [27]. Briefly, the shear stress-EI curve was linearized by plotting the reciprocal of EI as a function of the reciprocal of shear stress: 1/EI = [(SS1/2/EImax)(I/SS)] + 1/EImax, where SS is shear stress and EImax is the theoretical EI at infinite shear stress. The *x*-intercept of this line corresponds to the negative reciprocal value of shear stress causing half-maximal deformation (i.e., SS1/2); increased SS1/2 values indicate decreased RBC deformability.

### 2.4. Erythrocyte Aggregation Measurements

RBC aggregation was also determined by LORCA as described elsewhere [26]. The measurement is based on the detection of laser backscattering from the sheared (disaggregated) and then unsheared (aggregating) blood, performed in a computer-assisted system at 37°C. Backscattering data were evaluated by the computer and the aggregation index (AI), aggregation half time (*t*1/2), and aggregation amplitude (AMP) were calculated on the basis that there is less light backscattered from aggregating red cells. Aggregation measurements were determined using RBC in autologous plasma adjusted to 40% Hct and blood was fully oxygenated before the measurements.

### 2.5. Statistical Analyses

Continuous variables were expressed as mean ± standard deviation (SD), median (minimum–maximum values), and categorical variables as number and percent. Shapiro-Wilk tests were used for testing normality. If parametric test conditions were satisfied, Analysis of Variance (ANOVA) was used for comparisons among groups. The post hoc Tukey test was used when the ANOVA determined a significant difference. If parametric test conditions were not satisfied, the Kruskal-Wallis Variance Analysis was used for comparisons among groups. The post hoc Mann–Whitney *U* Test with Bonferroni Correction was used when the Kruskal-Wallis Variance Analysis determined a significant difference. For pairwise comparisons, if parametric test conditions were satisfied, Paired Samples *t*-test was used and if parametric test conditions were not satisfied, Wilcoxon signed rank test was used. All statistical analyses were done by SPSS 21.0. Power of the study was found to be over 95% with 95% confidence (*α* = 0.05).

## 3. Results

Table 1 shows the demographic characteristics and laboratory data of the groups. The study enrolled 13 newly diagnosed COPD patients (13 male) (mean age was 58.46 ± 9.72 years), 16 COPD patients with exacerbations (mean age was 67.75 ± 8.29 years), and 12 age and sex matched healthy controls (12 male) (mean age was 54.16 ± 11.4 years) (*p* > 0.05). The patients had a 48.8 ± 16.3 pack-years smoking history. RDW of COPD patients during exacerbations was higher compared to control and pretreatment of newly diagnosed patients groups (*p* = 0.0001 and *p* = 0.039, resp.), whereas fibrinogen level of this group was higher than all the other groups (*p* < 0.05). Additionally, Hb of COPD patients during exacerbations was less than posttreatment group (*p* = 0.05) and Hct of these patients were lower compared to both pre- and posttreatment groups (*p* = 0.003 and *p* = 0.05, resp.). There was no other statistically significant alteration between the parameters demonstrated in Table 1 (*p* > 0.05).

Table 2 shows pulmonary function test results of the subjects. Mean baseline predicted value of FEV1, FEV1/FVC, PEF, and FEF 25/75 of control group were significantly higher than COPD patients (*p* < 0.05). The treatment applied in newly diagnosed patients did not cause statistically significant alterations in pulmonary function test results. FVC (*p* = 0.001), FEV1, FEV1/FVC, and FEF 25/75 (*p* = 0.0001) of COPD patients with exacerbations were lower than control group while PEF of these patients were decreased compared to both control group and COPD patients receiving treatment (*p* = 0.0001 and *p* = 0.031, resp.).

Erythrocyte deformability (i.e., the elongation index, EI) for the RBC of the experimental groups was measured at nine shear stresses between 0.3 and 30.0 pascal (Pa) and shown in Table 3. RBC deformability of COPD patients with exacerbations measured at 0.53–30.00 Pa was decreased compared to control. On the other hand, EI of the same group measured at 0.30–9.49 Pa was lower than COPD patients receiving treatment (*p* < 0.05).

Table 4 demonstrates that EImax and SS1/2 of the exacerbation group were statistically significantly higher than the other groups (*p* < 0.05).

Erythrocyte aggregation parameters (AI and *t*_1/2_) are demonstrated in Table 5. AI (*p* = 0.0001) of COPD patients determined during exacerbations was higher whereas *t*_1/2_ (*p* = 0.001) was lower compared to control group. Increment of AI is in concordance with decrement of *t*_1/2_ and indicates augmentation of RBC aggregation.

## 4. Discussion

COPD is a leading cause of mortality and morbidity with increasing prevalence worldwide [28, 29]. It is a progressive condition characterized by a poorly reversible airflow limitation associated with an abnormal inflammatory response of the lung. There is evidence that, in different lung diseases, including COPD, blood cells may undergo distinct alterations. Erythrocytes are affected at different levels in COPD, including structural alterations [23, 30]. Furthermore, emerging themes in RBC biology support the new concept that erythrocytes are also signalling cells [31, 32], with important roles in the regulation of biochemical interactions with other cells in (patho) physiology. Hypoxia is one of the prognostic factors in COPD [21]. Tissue oxygenation depends on three factors: (1) the functional integrity of the pulmonary, cardiac, and vascular system, (2) the quantity and quality of the hemoglobin molecule and binding of oxygen to hemoglobin, and (3) the flow characteristics of the blood (hemorheology) [33, 34]. Blood flow, RBC aggregation, and deformability are main components of hemorheology. In the microcirculation, RBC must collapse to pass through narrow capillaries; hence, RBC deformability and aggregation are major determinants of resistance to flow. The ability of the entire RBC to deform is critically important to perform its function of oxygen delivery and is also a determinant of cell survival time in the circulation [35, 36]. RBC aggregation is a reversible process meaning a temporary linear or branched aggregate formation of the erythrocytes under critically low shear stress conditions. The physiological importance of erythrocyte aggregation which is the reversible adhesion of adjacent erythrocytes in circulation is its tendency to increase the blood viscosity in low shear flow and to disturb the passage in capillary circulation [37, 38].

Current study demonstrates hemorheological parameters (i.e., RBC deformability and aggregation) in newly diagnosed stage 1-2 COPD patients and the alterations in these parameters in response to one-month medical treatment and compares these parameters with values of COPD patients during acute exacerbations. RDW of COPD patients during exacerbations was increased, and Hb and Hct decreased as expected [39, 40]. No statistically significant alteration was observed in deformability of the RBC of the newly diagnosed experimental groups measured at nine shear stresses between 0.3 and 30.0 Pa as well as Elmax and SS1/2 values. Similar to our results, Karul et al. found no difference in osmotic fragility of stable COPD patients compared to controls [21]. On the other hand, Coppola et al. measured erythrocyte filterability in 15 COPD patients of 67–79 years old and found it decreased [20]. Santini et al. demonstrated an increase in membrane rigidity in erythrocytes of COPD patients with respect to controls. According to the authors, this increase in red cell rigidity might be due to enhanced sphingomyelin values [30]. Index of RBC deformability was also found to be decreased in chronic cor pulmonale decompensation [18, 22]. The disparity in RBC deformability results seems to be due to the technique used, age of the patients, severity of the disease, and the presence of complications. LORCA (laser assisted optical rotational cell analyzer) which is an up-to-date device to measure RBC deformability and aggregation with a good repetition of consecutive measurements was used in our study. Results of the current study demonstrate for the first time in the literature that RBC deformability of COPD patients with exacerbations is lower than both healthy subjects (at 0.53–30.00 Pa) and newly diagnosed COPD patients receiving treatment (at 0.30–9.49 Pa). It was mentioned before that impaired RBC deformability leads to increased SS1/2 values. The shear stress level of normal pulmonary circulation was demonstrated to be around 2-3 Pa [41]. This information increases the importance of the reduced RBC deformability determined at 1.69 and 3 Pa in COPD exacerbations, herein. When EImax and SS1/2 values were evaluated, erythrocyte deformability of the COPD patients with exacerbations was also found to be lower compared to COPD patients before treatment. These reports support the suggestion that presentation and comparison of data via this approach may provide a more comprehensive view of RBC stress-deformation behavior compared to shear stress-EI curves since it utilizes the entire range of shear stress [27]. The relations between pressure and blood flow become more complex in the microcirculation as the vessel diameter approaches average RBC size [42]. In addition to the existing lung pathology, decreased RBC deformability in COPD patients during exacerbations may serve as an unfavorable factor to worsen the clinical condition of the patient by further disrupting tissue oxygenation.

Another hemorheological parameter measured in our study is the RBC aggregation. The aggregation index (AI) and aggregation half time (*t*1/2) of newly diagnosed COPD patients were not different from healthy subjects. One month of appropriate treatment did not cause any statistically significant alteration in the aggregation parameters of the patients nor the FVC, PEF, and FEV_1_/FVC ratio. AI of COPD patients with exacerbations was higher whereas *t*_1/2_ was lower than healthy subjects. Increment of AI is in concordance with decrement of *t*_1/2_ and indicates augmentation of RBC aggregation. Similarly, Osipova et al. evaluated hemorheological properties by spontaneous aggregation of erythrocytes and observed a concurrently higher aggregation of erythrocytes in patients with COPD. The rheological blood properties worsened as hypertension of the small blood-circulation circle was increasing [43]. Index of RBC aggregation was found to increase in chronic cor pulmonale decompensation [18, 22]. Functional disturbances of erythrocytes in chronic bronchitis patients were shown to aggravate the course of the disease and need correction with membrane-stabilizing and membrane-repairing drugs [15, 18]. Plasma fibrinogen level is known as one of the most important determinants of RBC aggregation. The tendency to aggregate increases as fibrinogen concentration rises [34]. Thus, increased fibrinogen level observed in our COPD exacerbation group may provide an explanation for the augmentation of RBC aggregation in this group. Additionally, although not determined in our study, increases in plasma levels of inflammatory cytokines IL-6 and IL-8 were demonstrated in acute exacerbations of COPD [44] which may also alter RBC aggregation. It is also worth noting here that, in addition to decreased RBC deformability, increment of erythrocyte aggregation in COPD patients with exacerbation may also further play a role in diminishing circulation.

Oxidative stress represents one of the key mechanisms in the development of COPD [45]. Numerous studies have shown that oxidative stress is increased in lungs and systemic circulation of patients with COPD [46–50]. Markers of oxidative stress were found to increase even further during exacerbations of COPD [51] and in patients with very severe form of this disease [52]. Some studies have also demonstrated a marked decrease in plasma antioxidant capacity of COPD patients, while other studies have shown opposite findings [53]. RBC mechanical properties (i.e., erythrocyte deformability and aggregation) are known to be altered in response to oxidative stress [54, 55]. Although possible changes in oxidative stress parameters were not investigated in our study, due to the above information, it can be speculated here that impaired hemorheology herein may at least partly be due to increased oxidative stress in these patients.

## 5. Conclusion

In overview, the results of the current study indicate that exacerbations of COPD are characterized by significant hemorheological abnormalities known to adversely affect blood flow. The decrement observed in RBC deformability and the increment shown in erythrocyte aggregation during exacerbations might complement other pathophysiologic mechanisms, leading to worsening of oxygenation and thus clinical symptoms. The mechanisms of the hemorheological alterations could not be clarified. Oxidative stress and inflammation may be involved. Therapeutic approaches including antioxidants for normalizing hemorheological parameters during exacerbations of COPD may also be recommended. However, whether therapies to decrease RBC aggregation and enhance deformability directly and specifically can be developed and whether such therapies will relieve the patient's symptoms remain an area of active investigation. Further research with a larger number of patients to explain the mechanisms of hemorheological alterations may be necessary.

## Figures and Tables

**Table 1:** Laboratory data of the groups.

Parameter	Control group (*n* = 12)	Newly diagnosed patient group		Exacerbation group (*n* = 16)	*p*_1_	*p*_2_
		Before treatment (*n* = 13)	After treatment (*n* = 13)			
Hb (g/dl)	15.24 ± 1.75	15.54 ± 1.82	15.28 ± 1.51	**13.57 ± 2.2**7^*β*^	0.052	**0.033**
Hct (%)	44.95 ± 5.42	47.45 ± 6.17	45.78 ± 3.88	**40.93 ± 6.3**5^#,*β*^	**0.013**	**0.045**
RBC (m/*µ*L)	5.06 ± 0.61	5.21 ± 0.74	5.05 ± 0.55	4.57 ± 0.82	0.149	0.106
MCV (fL)	88.96 ± 3.73	91.41 ± 6.57	91.08 ± 5.81	90.13 ± 6.65	0.607	0.804
MCH (pg)	30.17 ± 0.97	29.98 ± 2.35	30.34 ± 2.07	29.83 ± 2.09	0.905	0.753
MCHC (g/dl)	33.95 ± 0.79	32.80 ± 1.07	33.32 ± 1.32	33.13 ± 1.41	0.060	0.246
WBC (K/uL)	7.41 ± 1.53	8.33 ± 1.85	8.66 ± 1.80	9.88 ± 5.40	0.228	0.231
Plt (K/uL)	214.64 ± 31.34	242.69 ± 49.77	242.38 ± 35.62	274.19 ± 154.62	0.263	0.175
Fibrinogen (mg/dL)	321.18 ± 115.24	312 ± 86.57	292.27 ± 74.51	**446.44 ± 193.6**5^*∗*,#,*β*^	**0.014**	**0.005**
RDW (%)	12.81 ± 0.77	13.99 ± 1.23	13.93 ± 1.34	**15.18 ± 1.6**2^*∗*,#^	**0.001**	**0.0001**

Values are expressed as means ± SD (Hb: hemoglobin, Hct: hematocrit, RBC: red blood cell, MCV: mean corpuscular volume, MCH: mean corpuscular hemoglobin, MCHC: mean corpuscular hemoglobin concentration, WBC: white blood cell, Plt: platelet count, and RDW: red blood cell distribution width). *p*_1_: difference between the control group, before treatment of newly diagnosed COPD, and the exacerbation group; *p*_2_: difference between the control group, after treatment of newly diagnosed COPD, and the exacerbation group; ^*∗*^*p* < 0.05: difference from control, ^#^*p* < 0.05: difference from before treatment of COPD, and ^*β*^*p* < 0.05: difference from after treatment of COPD.

**Table 2:** Pulmonary function test results of the subjects.

Pulmonary function test	Control group (*n* = 12)	Newly diagnosed patient group		Exacerbation group (*n* = 16)	*p*_1_	*p*_2_
		Before treatment (*n* = 13)	After treatment (*n* = 13)			
FVC (%)	97.67 ± 14.07	82.23 ± 13.97	88.77 ± 16.10	**68.5 ± 30.6**3^*∗*^	**0.005**	**0.006**
FEV_1_ (%)	99.25 ± 12.83	**66.69 ± 13.8**3^*∗*^	**75.54 ± 20.1**4^*∗*^	**64.36 ± 16.1**0^*∗*^	**0.0001**	**0.0001**
FEV_1_/FVC ratio	82.58 ± 6.04	**63.85 ± 4.7**1^*∗*^	**64.46 ± 8.6**6^*∗*^	**60.93 ± 8.1**3^*∗*^	**0.0001**	**0.0001**
PEF (%)	90.33 ± 12.06	**64.69 ± 16.1**2^*∗*^	**70.54 ± 24.5**6^*∗*^	**46.88 ± 29.3**8^*∗*,*β*^	**0.0001**	**0.0001**
FEF 25–75 (%)	90.67 ± 20.83	**35.38 ± 9.4**0^*∗*^	**42.08 ± 14.5**1^*∗*^	**25.38 ± 16.2**0^*∗*^	**0.0001**	**0.0001**

Values are expressed as means ± SD (FEV_1_: forced expiratory volume in the first second, FVC: forced vital capacity, PEF: peak expiratory flow, and FEF 25/75: forced expiratory flow 25% to 75%). *p*_1_: difference between the control group, before treatment of newly diagnosed COPD, and the exacerbation group; *p*_2_: difference between the control group, after treatment of newly diagnosed COPD, and the exacerbation group; ^*∗*^*p* < 0.05: difference from control, ^#^*p* < 0.05: difference from before treatment of COPD, and ^*β*^*p* < 0.05: difference from after treatment of COPD. *p*_1_ and *p*_2_ are findings via ANOVA whereas other symbols are findings by comparison of two groups.

**Table 3:** The EI values of the groups at different shear stresses.

Shear stress (Pa)	Control group (*n* = 12)	Newly diagnosed patient group		Exacerbation group (*n* = 16)	*p*_1_	*p*_2_
		Before treatment (*n* = 13)	After treatment (*n* = 13)			
0.30	0.06 ± 0.03	0.06 ± 0.02	0.05 ± 0.02	**0.04 ± 0.0**1^#^	**0.039**	0.333
0.53	0.12 ± 0.05	0.12 ± 0.05	0.08 ± 0.04	**0.05 ± 0.0**1^*∗*,#^	**0.010**	**0.001**
0.95	0.21 ± 0.08	0.21 ± 0.08	0.17 ± 0.057	**0.12 ± 0.0**2^*∗*,#^	**0.002**	**0.001**
1.69	0.33 ± 0.10	0.31 ± 0.09	0.26 ± 0.07	**0.22 ± 0.0**2^*∗*,#^	**0.002**	**0.001**
3.00	0.41 ± 0.07	0.40 ± 0.08	0.36 ± 0.06	**0.32 ± 0.0**2^*∗*,#^	**0.001**	**0.001**
5.33	0.49 ± 0.05	0.47 ± 0.06	0.45 ± 0.06	**0.42 ± 0.0**2^*∗*,#^	**0.001**	**0.001**
9.49	0.55 ± 0.04	0.54 ± 0.05	0.51 ± 0.05	**0.50 ± 0.0**2^*∗*,#^	**0.001**	**0.003**
16.87	0.60 ± 0.04	0.59 ± 0.04	0.57 ± 0.04	**0.55 ± 0.0**2^*∗*^	**0.006**	**0.002**
30.00	0.63 ± 0.04	0.63 ± 0.04	0.61 ± 0.04	**0.59 ± 0.0**2^*∗*^	**0.007**	**0.017**

Values are expressed as means ± SD; EI: elongation index and Pa: pascal. *p*_1_: difference between the control group, before treatment of newly diagnosed COPD, and the exacerbation group; *p*_2_: difference between the control group, after treatment of newly diagnosed COPD, and the exacerbation group; ^*∗*^*p* < 0.05: difference from control, ^#^*p* < 0.05: difference from before treatment of COPD, and ^*β*^*p* < 0.05: difference from after treatment of COPD. *p*_1_ and *p*_2_ are findings via ANOVA whereas other symbols are findings by comparison of two groups.

**Table 4:** The EImax and SS1/2 values of the groups.

	Control group (*n* = 12)	Patient group (*n* = 13)		Exacerbation group (*n* = 16)	*p*_1_	*p*_2_
		Before treatment	After treatment			
EImax	0.64 ± 0.21	0.68 ± 0.24	0.66 ± 0.37	**0.83 ± 0.1**5^*∗*,#,*β*^	**0.001**	**0.0001**
SS1/2	2.06 ± 1.29	3.16 ± 3.65	4.64 ± 7,92	**6.17 ± 3.**3^*∗*,#,*β*^	**0.0001**	**0.0001**

Values are expressed as means ± SD; EImax: theoretical EI at infinite shear stress and SS1/2: shear stress at half-maximal deformation. *p*_1_: difference between the control group, before treatment of newly diagnosed COPD, and the exacerbation group; *p*_2_: difference between the control group, after treatment of newly diagnosed COPD, and the exacerbation group; ^*∗*^*p* < 0.05: difference from control, ^#^*p* < 0.05: difference from before treatment of COPD, and ^*β*^*p* < 0.05: difference from after treatment of COPD. *p*_1_ and *p*_2_ are findings via ANOVA whereas other symbols are findings by comparison of two groups.

**Table 5:** The RBC aggregation measurements of the groups.

	Control group (*n* = 12)	Newly diagnosed patient group		Exacerbation group (*n* = 16)	*p*_1_	*p*_2_
		Before treatment (*n* = 13)	After treatment (*n* = 13)			
AMP (au)	25.0 ± 2.91	25.1 ± 3.90	24.65 ± 3.18	20.77 ± 3.59^*∗*,#,*β*^	**0.002**	**0.002**
AI (%)	68.15 ± 5.90	72.33 ± 6.16	73.16 ± 8.17	79.96 ± 3.61^*∗*^	**0.0001**	**0.007**
*t*1/2 (sn)	1.79 ± 0.55	1.45 ± 0.55	1.39 ± 0.68	1.01 ± 0.37^*∗*^	**0.001**	**0.003**

Values are expressed as means ± SD; AMP: amplitude of aggregation; AI: aggregation index; *t*1/2: aggregation half time. *p*_1_: difference between the control group, before treatment of newly diagnosed COPD, and the exacerbation group; *p*_2_: difference between the control group, after treatment of newly diagnosed COPD, and the exacerbation group; ^*∗*^*p* < 0.05: difference from control, ^#^*p* < 0.05: difference from before treatment of COPD, and ^*β*^*p* < 0.05: difference from after treatment of COPD.
